# Changes in sobriety in the Swedish population over three decades: age, period or cohort effects?

**DOI:** 10.1111/j.1360-0443.2011.03692.x

**Published:** 2012-04

**Authors:** Kozma Ahacic, Robert F Kennison, Ingemar Kåreholt

**Affiliations:** 1 Social Medicine, Department of Public Health Sciences, Karolinska InstitutetStockholm, Sweden; 2 Department of Psychology, California State UniversityLos Angeles, CA, USA; 3 Aging Research Center, Karolinska Institutet and Stockholm UniversityStockholm, Sweden

**Keywords:** Abstainer, abstinence from alcohol, age, alcohol consumption, APC-analysis, cohort, period, Sweden, trends

## Abstract

**Aims:**

This study aimed to examine age, cohort and period trends in alcohol abstinence.

**Design:**

Two surveys, the Level of Living Survey collected in 1968, 1974, 1981, 1990 and 2000, and the Swedish Panel Study of the Oldest Old (SWEOLD) collected in 1992 and 2002, were studied with graphical depictions of cross-sectional and longitudinal data presented over time and over age. Cross-sectional 10-year age group differences, time-lag differences between waves and within-cohort differences between waves for 10-year birth cohorts were examined. Logistic regression models were applied to confirm the observed patterns.

**Setting:**

The samples were representative of the Swedish population.

**Participants:**

Participants ranged in age from 18 to 75 (*n* = 5000 per wave), and 77+ at later waves (*n* = 500).

**Measurements:**

Alcohol abstinence was determined by asking ‘Do you ever drink wine, beer, or spirits?’, where a ‘no’ response indicated abstinence.

**Findings:**

Decreases in abstinence rates were observed from 1968 to 2000/02. While cross-sectional analysis indicated increased abstinence with advancing age, the longitudinal analysis suggested otherwise. Inspection of cohort differences revealed little change within cohorts and large differences between cohorts; abstinence rates declined in later-born cohorts up to the 1940s birth cohorts; stability was observed in cohorts born since the 1940s. Logistic regression models indicated that neither age nor period were significant (*P* > 0.05) predictors of abstinence when cohort (*P* < 0.001) was included.

**Conclusion:**

Decreasing proportions of total alcohol abstainers in Sweden from 1968 to 2000 appear to be attributable primarily to decreases in successive cohorts rather than drinkers becoming abstainers.

## INTRODUCTION

The purpose of this study is to examine age, cohort and period effects in alcohol abstinence rates for the Swedish population from 1968 to 2002. Whereas age effects are due to biological, psychological and behavioural processes connected with maturation and ageing [1], cohort effects reflect historical differences in the social or physical environment that occur during development in childhood and young adulthood [2]. Period effects refer to the events and influences during a specific time-period, such as the period from 1968 to 2002. Both cohort and period effects may result from the same kind of sudden events and changes in the physical and social environment (e.g. policy changes), but they may also reflect the influences from more subtle changes such as shifting attitudes. Despite their possible common origin, it is important to differentiate between cohort and period effects in order to place events and influences into a proper time-frame.

Sweden is a country with restrictive laws on the sale of alcohol, high consumption taxes and other measures aimed to restrict the use of alcohol in public locations. These policies enjoy substantial public support to this day [3]. In the past, Sweden curbed alcohol use by implementing an alcohol rationing system, the so-called Bratt system, which was gradually rolled out during the 1910s and remained in effect until 1955 [4]. The cohorts most affected by this rationing system are now reaching old and very old age.

The rationing of alcohol employed under the Bratt system was extensive, but it was also applied selectively by favouring older males in higher socio-economic groups with larger rations [4]. Women, younger people and the unemployed had difficulty obtaining any rations at all. Ultimately, this system was at odds with the developing social egalitarian views of the Swedish government and its people. In addition, the efficacy of the programme was questionable. Abstinence rates decreased under the Bratt system, which was one of the explicit reasons why the government abandoned it [5]. However, alcohol consumption rates continued to increase in the years after its abolishment [6] suggesting that, while not promoting abstinence, it may have reduced alcohol consumption.

Based on these historical changes, it seems likely that there may be cohort effects in abstinence rates. Specifically, it is hypothesized that later-born cohorts will display lower alcohol abstinence rates compared to earlier-born cohorts.

However, other developmental trends may exist that may complicate the issue. For example, there may exist age-related changes in alcohol consumption. In several countries adult alcohol consumption has been shown to decrease with age. One apparent reason for this is the increase in health problems that occur with age. This, combined with the deleterious effects of heavy drinking over time, is likely to cause people to decrease their drinking, abstain completely or experience greater morbidity and mortality.

A clear understanding of the trends in drinking behaviour remains elusive, because studies have generally not disentangled cohort and age effects in their data (but see [7–10]). In order understand alcohol consumption trends it is important to also examine trends for both heavy drinkers and abstinence separately from average consumption rates.

The relationship between alcohol consumption and health is complex, often characterized as a J-shaped function, in which small to moderate consumption of alcohol is considered beneficial while heavy consumption is known to have deleterious effects on health and other factors [11–13]. Although there has been considerable attention paid in the scientific literature to heavy drinking and its associated health outcomes, comparably less attention has been given to abstinence, where even some basic epidemiological knowledge is lacking.

To our knowledge this is the first study to apply an age, period and cohort approach to the study of prevalence of abstinence from alcohol. In the study age, period and cohort patterns in abstinence rates will be examined.

## METHODS

The data used in this study were from two Swedish studies. The Level of Living Surveys (LNU) were conducted in 1968, 1974, 1981, 1991 and 2000 [14]. All waves were nationally representative cross-sectional samples (*n* = 5000 per wave) of the Swedish adult population and included people aged 15–75 years. In the 1991 wave the lower age limit was raised from 15 to 18. Between 1968 and 2000 the response rate fell from 90.6% (*n* = 5654) to 76.6% (*n* = 5126). In 1968 the sample consisted of approximately 6000 people between the ages of 15 and 75. When the survey was repeated in 1974, participants from the initial sampling in 1968 who were still living in Sweden and under the age of 76 were recruited to participate. In 1974 and in successive waves, samples from later-born birth cohorts and recent immigrants were added to maintain the sample's cross-sectional representativeness.

The Swedish Panel Study of the Oldest Old (SWEOLD) study, originating from LNU, included participants aged 77–99 [15]. It consists of two nationally representative cross-sectional samples (*n* = 500 per wave). Interviews were conducted in 1992 and 2002 with response rates of 95.4% and 88.5%, respectively. Each of the two waves included all those people aged 77+ who were originally eligible for at least one wave of the LNU. Participants were interviewed in their current residence, and those who could not be interviewed directly were interviewed by proxy (11.9 and 12.8% for 1992 and 2002, respectively).

The interviews in both studies included questions about alcohol consumption. People who answered ‘no’ to the question ‘Do you ever drink wine, beer (4.5% alcohol) or spirits?’ were coded as abstainers.

### Analysis

A graphic approach was used to identify age, period and cohort effects in the extant data of this study. Two data patterns that demonstrate unequivocal developmental trends may be identified [16]. First, when no effects are present no measurable differences, neither cross-sectional, longitudinal nor time-lag differences, should be found. Secondly, when only one effect is operating, then two of the measurable differences will agree and the third will not be detectable. For example, an age effect without period or cohort effects should result in similar cross-sectional and longitudinal patterns, while time-lag differences will be negligible.

Cross-sectional differences were studied with 10-year age groupings measured in 1968, 1974, 1981, 1991/1992 and 2000/2002. Time-lag differences were identified between waves for 10-year age groups. Within-cohort differences were computed between waves for 10-year birth cohort panels followed over time.

Identification of age, cohort and period effects necessitates alternate graphic representations of these differences. Longitudinal differences are plotted in two formats: over years, i.e. calendar time, and over age. All three measurable differences have such alternatives, yielding a total of six different graphic descriptions.

Because irregular intervals between waves exist, some waves were associated with truncated cohorts, whereby data were omitted in the plots for the 1965–1974 cohort in the 1998 and 1991 waves; for the 1955–1964 cohort in 1974; for the 1945–1954 cohort in 1968; and for the 1925–1934 cohort in 2002. Similarly, data for the oldest-aged 10-year cohorts was discarded because high attrition/mortality rates were observed. This occurred for the 1895–1904 cohort in 1974 and 2002 and for the 1892–1894 cohort in 1974 and later.

The irregular intervals also made it necessary to approximate some of the data points for the graphs displaying cohort differences by age. The estimated data points were produced by computing curves describing the age differences in the longitudinal design, i.e. estimates for the specific ages were taken from the Excel spreadsheet used for the longitudinal age curves.

To examine whether longitudinal and time-lag differences were negligible, two logistic regression models were computed. The first included cohort and period variables, and the second included cohort and age variables. Based on the graphic results, where linear effects were observed, the age and cohort measures were treated as linear functions in the regression models. A third model included only cohort, which was modelled for both linear and curvilinear (quadratic) changes. Controls for truncated cohorts (dummy variables for the cohorts in the waves enumerated above) and age groups below age 18 (dummies for these ages in the 1968, 1974 and 1981 wave) also were included. To model all period contrasts, i.e. the odds ratio between the two SWEOLD surveys, also necessitated a control dummy variable contrasting SWEOLD with LNU. When cohort differences were controlled for statistically, age and period effects were not significant. This finding, when taken together with the graphic results, shows that no age–period–cohort model is warranted to disentangle the effects.

## RESULTS

**Figure 1:**
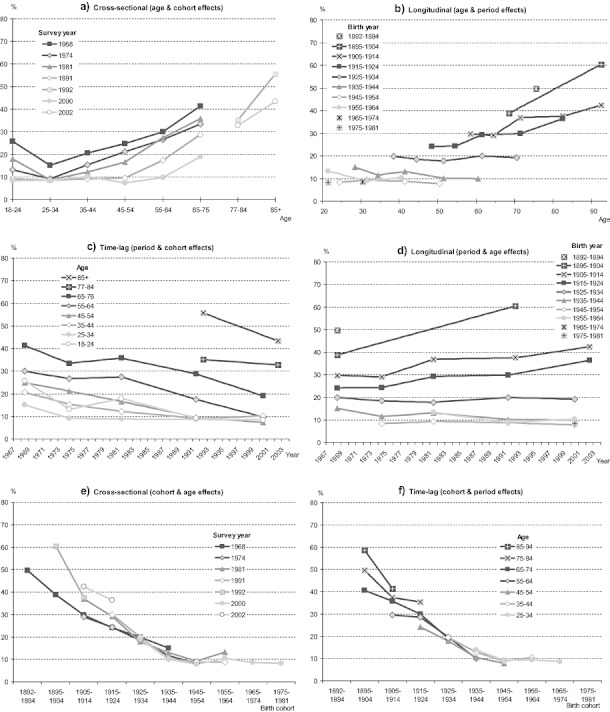
Percentage of abstainers. Cross-sectional, time-lag and longitudinal analysis of age, period and cohort patterns

Figure 1 shows the abstinence rates, where age plots appear in the top row; period plots appear in the middle row; and cohort plots appear in the bottom row. The two confounded effects, whether age, cohort or period, are identified in the heading of each graph. Figure 1a,e displays the cross-sectional differences; Fig. 1c,f shows the time-lag differences; and Fig. 1b,d displays the longitudinal differences.

### The alternate descriptions of the age differences

Figure 1a displays the data organized cross-sectionally. The individual lines indicate abstinence rates for the different age groups and waves. Aside from an initial decline in abstinence rates exhibited between the 18–24 age group and the 25–34 age group, abstinence rates were generally higher for successively older age groups. Taken together, the cross-sectional age differences display a curvilinear age relationship, whereby abstinence rates increased for each successively older age group.

Figure 1b shows the longitudinal age differences. Each line follows a single cohort displayed over age. For example, the 1925–1934 cohort is followed from ages 38 to 71 in four increments, each varying in duration. For that cohort, the changes in abstinence rates are small, at best, suggesting relative stability. While earlier-born cohorts began with higher abstinence rates compared to later-born cohorts, generally those rates remained stable as the cohort ages. After age 65 abstinence rates showed small age-related increases in the earliest-born cohorts. The overarching pattern, however, does not indicate any clear age-related changes in abstinence rates.

### Period differences

Figure 1c shows the time-lag differences. The individual lines show abstinence rates within age groups across waves. The percentage of participants reporting abstinence decreased from 1968 to 2000/2002 in all age groups. The largest decreases (in terms of percentage) occurred in the older age groups, which had the highest rates of abstinence.

Figure 1d presents the period differences. The data are those data that appear in the plot shown in Fig. 1b, but organized over time rather than over age. The observed pattern indicates that initial abstinence rates in 1968 changed little over the time-period. If anything, abstinence rates increased somewhat in some of the cohorts from 1968 to the most recent testing.

### Cohort differences

Figure 1e shows the cross-sectional cohort differences. The differences are similar to those observed in Fig. 1a, but transposed and distributed over cohorts instead of over age. The lines show the abstinence rates between cohorts. Earlier-born cohorts had higher abstinence rates compared to later-born cohorts. This pattern is observed for all waves. Taken together, the cohort trends of the different survey years show a curvilinear trend. One exception to this observation occurs between surveys for the earliest-born cohorts, where some disparity is possibly indicated (with a higher prevalence for SWEOLD) by lines that do not coincide completely.

Figure 1f shows the time-lag cohort differences, allowing for comparison of cohorts' abstinence rates at similar ages. At each observed age, earlier-born cohorts had higher abstinence rates compared to later-born cohorts. This pattern is demonstrated for all ages. When viewed as a whole, the cohort trends viewed over age suggest a curvilinear relationship, which is similar to the observed cohort dependencies seen in the cross-sectional cohort differences displayed in Fig. 1e. There is also some possible disparity among the earliest-born cohorts.

In sum, the cross-sectional and time-lag trends found for the different cohorts were generally consistent, suggesting that the prevalence of participants reporting abstinence was due to cohort effects. Earlier-born cohorts generally had higher abstinence rates than later-born cohorts.

### Logistic regression analyses

The results of the logistic regression models appear in Table 1. The bivariate models were fitted with the unadjusted predictors of age, period and cohort, respectively. Model 1 shows that the period differences were not significant (*P* > 0.05) when adjusted for cohorts. Model 2 shows that age differences were not significant (*P* > 0.05) when adjusted for cohorts. In order to describe more clearly the curvilinearity observed for the cohorts, a square term was added to the last model of the cohort effect.

**Table 1:** Logistic regression show odds ratios (ORs) for abstaining from alcohol in the adult Swedish population between 1968 and 2002, for cohort (birth year), time-period (survey wave) and age.

	*Bivariate*	*Model 1*	*Model 2*	*Model 3*
				
	*OR*	*OR*	*OR*	*OR*
Cohort				
Linear^a^	0.75***	0.72***	0.74***	0.60***
Square	–	–	–	1.02***
Time-period^b^				
1968	2.86***	1.03	–	–
1974	2.05***	0.89	–	–
1981	1.97***	1.07	–	–
1991/1992	1.32***	0.99	–	–
2000/2002	Ref	Ref	–	–
(1992)^c^	1.24	0.90	–	–
(2002)^d^	Ref	Ref	–	–
Age				
Linear^e^	1.28***	–	0.98	–
Pseudo^f^*R^2^*		*0.13*	*0.13*	*0.12*

* *P* < 0.05

** *P* < 0.01;

*** *P* < 0.001.

a The increase of odds for an increase of 10 years in time for when cohorts were born.

b The 2000/02 wave is the reference category (OR 1.0). Age groups 15–18 are controlled for in the models, to ensure that the same age range is compared across the Level of Living Surveys (LNU) surveys.

c Control, the 1992 survey concerned age 77+, whereas earlier waves concerned ages 15–76. This is an interaction term showing the OR between the age group 77+ in 1992 and the same age group in 2002 (Ref).

d The reference (OR 1.0) for the contrast between the two Swedish Panel Study of the Oldest Old (SWEOLD) surveys. To model the OR between the two SWEOLD surveys it is also necessary to contrast SWEOLD with LNU. This comparison between the age group 77+ in 2002 and the age group 18–76 in the 2000 wave was omitted in the table, as it does not reflect period differences but all kinds of differences between the LNU and SWEOLD surveys including those connected to age. For this contrast the non-adjusted or bivariate OR was 5.09*** and the adjusted 1.69***.

e The increase of odds for an increase of 10 years in age.

f McKelvey & Zavoina pseudo-*R*^2^ gives a fair estimate of the model fit [36].

### Sex differences

**Figure 2:**
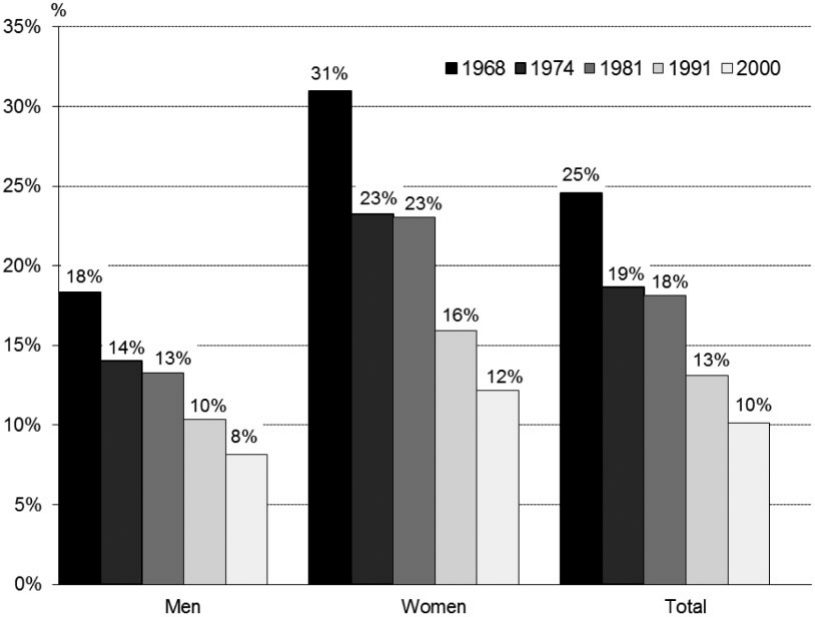
Percentage of abstainers in the Swedish population aged 18–75 years in 1968, 1974, 1981, 1991 and 2000

Separate analyses conducted for males and females indicated that similar patterns of effects for the two groups. The only difference is that the odds for abstinence were lower for men than for women [odds ratio (OR) = 0.56, *P* < 0.001]. Figure 2 shows the decrease of the abstinence rates in the Swedish population aged 18–75 between 1968 and 2002 by gender.

## DISCUSSION

As indicated by the different graphical displays, abstinence rates were dominated by cohort effects. Rates of abstinence decreased in a curvilinear manner for later-born cohorts. While approximately half the 1895–1904 cohort was abstinent, the rate of abstinence decreased successively to 1/10th of the 1945–1954 cohort. Cohorts born after the 1945–1954 cohort had approximately the same rate of abstinence.

There was little evidence for age or period effects between 1968 and 2000/2002. All cohorts had aged with little change while simultaneously exhibiting these large between-cohort differences. Over time, earlier-born cohorts with higher abstinence rates were replaced by later-born cohorts with lower abstinence rates. That there were no age effects indicated for abstinence was contrary to the suggestion of an earlier cross-sectional analysis of age differences [17].

The observed cohort effects may not only have caused erroneous inferences about age; there may also have been wrong conclusions about period effects. According to a government report, in 1946 the abstinence rate was estimated to be 12% among men and 36% among women, and in 1967 the corresponding rates were 12% and 28% [18]. Our results show that in ages 18–75 the percentage of abstainers decreased from 18% to 8% among men and from 31% to 12% among women, confirming earlier analysis [4]. While explanations for decreasing abstinence rates may have been attributed mistakenly to period effects, there remains the unlikely possibility that such effects could have occurred. Although abstinence rates had decreased over the last three decades of the 20th century, our results suggest that the changes in abstinence rates were due solely to the cohort replacement.

Similar to the US ‘baby boomer’ generation, Swedish people born in the 1940s have been identified as a generation that changed society [19]. During the 1970s, the popular youth culture was associated with going out to bars, clubs and restaurants, where alcohol consumption was standard fare. Consequently, it has been argued that a ‘conscientious generation’ for people reaching adulthood during the 1930s was replaced by a ‘hedonistic generation’ of people reaching adulthood during the 1970s [17,20]. These later-born cohorts were reported to have rejected earlier generations' attitudes of collectivism and self-discipline. This change in values was a kind of counter manifestation, and a ‘generation gap’ ensued with the time of youth. While there might be some truth to this hypothesis, our results suggest that it lacks corroborating evidence. The present data show that abstinence rates were decreasing up to the 1940s generation, but not for later generations.

In our analysis, abstinence rates stand out as a cohort-bound phenomenon. Abstinence from alcohol seems to develop early, during adolescence and early adulthood, and then it remains relatively constant across the adult life-course. Alcohol rationing coincided with adulthood for cohorts that demonstrated decreasing abstinence rates. This cohort effect is likely to reflect changes in public opinion, which occurred decades before baseline.

It seems likely that the observed cohort effects were caused, in part, by changes in Swedish policy on alcohol sales and distribution, but the observed changes in abstinence rates also run concurrently with the modernization and secularization of Swedish society. The cohorts born in the first half of the 20th century were born during a time-period when the social democrats gained power and a socialist model was adopted. The working-class movement, sports movement and non-conformist religious revival movement are often said to have formed modern Swedish society during these years. These movements were intertwined with the temperance movement, and abstinence was often an objective, or at least an issue, raised by these movements [21,22].

Based on the US and continental models, it was heavy consumption of alcohol and high rates of alcoholism during the 19th century that paved the way for the Swedish temperance movement. In Sweden the temperance movement reached its peak in the 1920s, and culminated in a public vote for alcohol prohibition, but total prohibition was never realized because the Bratt system reflected a compromise between those who wanted to total prohibition and those who did not [22]. It seems likely that these cohort differences, i.e. decreasing abstainer rates, may be viewed as the aftermath to the political struggle around this issue.

The cohort differences observed in our data were of similar magnitude from 1968 to 2002. Abstinence rates decreased into the mid-1950s and then stabilized thereafter. This most probably reflects changes in drinking behaviour for people born around 1940. It was this cohort and later cohorts that were affected by abolishment of the Bratt system. Thus, our results indicate that abstinence rates may be influenced by government interventions.

Abstinence rates seem to vary considerably between countries [23]. Because it is not possible to identify clearly age, period or cohort effects based on the incomplete information provided by earlier studies, many of these earlier studies remain uninformative in this respect. This complicates speculation about possible patterns elsewhere. If one accepts that cohort effects may play a major role in abstinence patterns, then these patterns are likely to differ between countries (as indicated by cross-sectional age-relatedness studies [7,^24^–27]. To our knowledge, there has been no evidence of similar cohort effects in the United States, which has had a similar history [25]. Finland has also had a similar history to Sweden, and in that country abstinence rates were shown to have decreased from 1946 to 1976 [28,29]. A better understanding of age, cohort and period effects in alcohol abstinence rates in other countries such as Finland and the United States would greatly facilitate a broader understanding the causes of alcohol consumption patterns.

Not surprisingly, the observed abstinence patterns do not coincide with the pattern of alcohol related mortality reported in Sweden [30]. Age, period and cohort patterns are likely to differ based on which measures of alcohol consumption are studied, e.g. abstinence, average consumption, alcohol abuse or the consequences of this consumption, such as morbidity or mortality. For example, changes in health and medicine are likely to have strong influences on mortality, and these changes run semi-independently to alcohol abstinence or consumption rates.

Our results demonstrate trends in abstinence at a macro level and we do not address patterns of abstinence within individuals. To address this issue, an analysis of panel data from 1968 to 1991 was conducted [17]. Approximately half the self-identified abstainers in 1991 had not reported abstinence at baseline in 1968. This suggests that individuals are likely to change over time; but the study's results also indicated no major change in correlates of abstinence over time.

By using a combination of the graphic layouts and modelling, we have approached the age, period and cohort dilemma for a variety of outcomes in the LNU and SWEOLD data material earlier [31–34]. Unfortunately, there remain issues that potentially complicate interpretation of our results. For example, there are likely to be problems associated with measurement, non-response and changes in representativeness over time and age. While it is true that alcohol consumption is related to increased mortality risk, the influence of changes in selective mortality on the results is not likely to have been substantial. However, it seems likely that LNU underestimated abstinence and the cohort differences in abstinence between the older cohorts. In our analysis, the SWEOLD studies had higher abstinence rates than LNU, even when adjusted for the cohort differences. These surveys, including the oldest aged 76+, also had higher response rates than LNU, and a design specifically targeting to include the hospitalized and institutionalized part of the population, among other methods by using proxy interviews. This is also likely to represent a part of the population with higher abstinence rates. Earlier study confirms that non-responders may be more abstinent [35].

The observed large cohort effects demonstrate the importance of early-life determinants for alcohol-related health behaviour in adult and later life.

### Declarations of interest

None.
